# Enhancing Rats’ Diet with Ethyl Esters Derived from Linseed Oil and Examining the Resulting Changes in Their Blood Fatty Acid Profile

**DOI:** 10.3390/ijms252413668

**Published:** 2024-12-20

**Authors:** Ewa Sokoła-Wysoczańska, Katarzyna Czyż, Anna Wyrostek

**Affiliations:** 1 The Lumina Cordis Foundation, Szymanowskiego 2a, 51-609 Wrocław, Poland; 2 Institute of Animal Breeding, Wrocław University of Environmental and Life Sciences, Chełmońskiego 38c, 51-630 Wrocław, Poland

**Keywords:** linseed, ethyl esters, fish oil, erythrocytes, serum, alpha-linolenic acid—ALA, eicosapentaenoic acid—EPA, docosahexaenoic acid—DHA

## Abstract

Omega-3 fatty acids are an important factor contributing to the prevention and cure of numerous diseases, and therefore their supplementation with diet is a significant issue. There are numerous supplements on the market containing omega-3 acids, of both plant and animal origin. In our study, we compared an effect of linseed oil, ethyl esters of linseed oil and fish oil supplementation to rats’ diet on their blood serum and erythrocyte fatty acid profile. The animals were divided into nine groups, three groups did not receive supplements and differed in dietary fat content, three were fed a high-fat diet for the whole experiment and supplemented, three were fed a high-fat diet and then the control one together with supplements. The experiment lasted 12 weeks. Significant changes in the profile of omega-3 fatty acids, as well as the ration of omega-6 to omega-3, were found in supplemented groups compared to the controls, the changes were more beneficial in groups supplemented with ethyl esters of linseed oil. The results were also more beneficial in groups where in addition to supplementation, there was also a dietary change from high fat to control during the supplementation period. In the case of the erythrocytes, the alpha-linolenic acid (ALA) content in ethyl ester-supplemented groups increased (*p* < 0.05) by about 60–120%, eicosapentaenoic acid (EPA) by 80%, and docosahexaenoic acid (DHA) by 41–60% compared to the control. In turn, in the blood serum, this increase (*p* < 0.05) was about 70–125%, 26–40%, and 38–54%, respectively. In the case of ethyl esters, except for an increase in EPA and DHA acids, higher values of ALA were found, which can be further used in the process of conversion to longer-chain omega-3.

## 1. Introduction

The group of polyunsaturated fatty acids (PUFAs) includes omega-3 and omega-6 acids, and their main representatives are linoleic acid (LA, omega-6) and alpha-linolenic acid (ALA, omega-3). The role of ALA is of particular importance for both humans and animals as it is a precursor of other acids from the omega-3 family, eicosapentaenoic acid (EPA) and docosahexaenoic acid (DHA). Both groups of acids differently affect the metabolic functions of the organism; while omega-3 acids exhibit an anti-inflammatory activity, omega-6 are responsible for pro-inflammatory reactions in the body [1].

In the so-called Paleolithic diet, alpha-linolenic acid (ALA), in addition to linoleic acid (LA) from the omega-6 family and oleic acid (OA) from the omega-9 group, is used as the basic component of consumed fats, and the ratio of these acids was beneficial from a health point of view [2]. Nowadays, these acids are no longer commonly present in diets and their proper balance is disturbed. According to numerous scientific data, the ratio of omega-6 to omega-3 should be 4.5:1 to 10:1, while currently in the Western diet, the ratio is 15–16:1 or even 20:1 [3,4,5,6,7,8]. This situation is believed to be one of the reasons for the development of many chronic diseases, including the so-called civilization diseases like cancers, diabetes, obesity, degenerative diseases, metabolic diseases, cardiovascular problems, atherosclerosis, arteriosclerosis, ischemic heart diseases, immune system problems, allergies, dermatological diseases, gastrointestinal problems, hormonal problems, mental problems, memory disorders, Alzheimer’s disease, Parkinson’s, and emotional problems such as depression, aggression, and hyperactivity in children (ADHD) [9,10,11].

Referring to the anti-inflammatory properties of omega-3 fatty acids, scientific evidence from recent years has led to some changes in the understanding of chronic disease, which is related to the nature of inflammation. The literature reports reveal that chronic low-grade systemic inflammation is a factor leading to most chronic diseases over many years [12,13,14]. Therefore, an increase in the level of ALA in the diet may at the same time reduce the activity of omega-6 acids exhibiting pro-inflammatory properties, and increase the level of EPA and DHA acids exhibiting anti-inflammatory properties, also restoring the proper ratio of these groups of acids [15,16,17]. One of the richest sources of alpha-linolenic acid is linseed (*Linum usitatissimum* L.) and the oil manufactured from it; therefore, this plant has attracted much attention as a dietary supplement [1,18,19]. However, in addition to a profitable fatty acid profile, linseed and linseed oil also contain some unprofitable ingredients like linatine, cyanogenic compounds, or phytic acid [1], which may be the cause of some concern among consumers and to some degree, limit its application. The solution for this may be supplementation with ethyl esters of linseed oil, which are free of these harmful substances, and the effectiveness of which we have demonstrated in previous studies [5,6,7,8]. Considering fish oil consumption as a source of EPA and DHA, the literature reports that this can be associated with some risk due to the presence of environmental toxins, e.g., mercury, dioxins, polychlorinated biphenyls, or hypervitaminosis associated with high levels of fat-soluble vitamin D and A [9,20,21]. In addition, plant-based diets are becoming increasingly popular for various health, ethical, and environmental reasons [22].

The aim of this study was to examine the effect of rats’ diet supplementation with linseed oil ethyl esters, compared to raw linseed oil and fish oil, on the fatty acid profile of rat blood serum and erythrocytes, also considering the diet fed to the animals.

## 2. Results

### 2.1. Body Weight

Table 1 presents the results of body weight of the examined animals after the acclimatization period, at the beginning of supplementation and at the end of the experiment. Despite some differentiation in the values obtained, no statistically significant differences were noted between the groups in the case of body weight measurements after the acclimatization period and at the beginning of supplementation. However, at the end of the experiment, significant differences were observed between the groups, as presented in Table 1.

No disease symptoms like, e.g., diarrhea were observed during the whole period of this study.

### 2.2. Fatty Acids Composition of Erythrocytes

**Figure 1:**
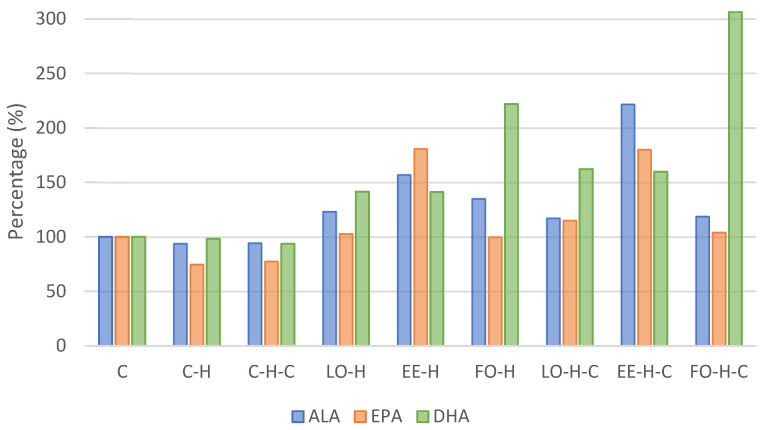
Omega-3 acids content in experimental group erythrocytes relative to the control group (C). Explanations of groups–see Table 1; ALA–alpha-linolenic acid; EPA—eicosapentaenoic acid; DHA—docosahexaenoic acid.

The results concerning the fatty acid profile in erythrocytes of the experimental animals are presented in Table 2 and in Figure 1.

Considering saturated fatty acids (SFAs), in general, the highest content was found for palmitic acid (C16:0), followed by stearic acid (C18:0). The content of palmitic acid was the highest in group C-H-C and it was significantly higher compared to groups C, EE-H, LO-H-C, and FO-H-C (*p* < 0.05). In turn, the level of stearic acid was the highest in group LO-H and it differed significantly from groups C, LO-H-C, EE-H-C, and FO-H-C (*p* < 0.05). Statistically significant differences between the groups were also observed in the case of other SFAs, i.e., lauric acid (C12:0), the content of which was the highest in group C-H and differed significantly from groups C, FO-H, LO-H-C, and FO-H-C (*p* < 0.05), margaric acid (C17:0) with the highest content in groups C-H and FO-H, statistically higher compared to groups C, LO-H-C, and FO-H-C (*p* < 0.05), arachidic acid (C20:0) that was the highest in group FO-H-C and statistically different from groups C-H and C-H-C (*p* < 0.05), behenic acid (C22:0) for which the level was the highest in group LO-H-C and differed from all control groups and those fed a high-fat diet for the whole experiment (*p* < 0.05), as well as lignoceric acid (C24:0) which was found on the highest level in group FO-H and differed statistically from the control groups, LO-H, LO-H-C, and FO-H-C (*p* < 0.05) (Table 2).

Changes in the content of individual saturated fatty acids are reflected in their total content in analyzed samples, which was the highest in group LO-H and differed statistically from groups C and FO-H-C (*p* < 0.05) (Table 3).

In the case of unsaturated fatty acids (UFAs), the highest shares in the total pool of fatty acids were obtained for oleic acid (C18:1n9c), followed by linoleic acid (C18:2n6c), and arachidonic acid (C20:4n6). The content of oleic acid was the highest in group C and differed significantly from groups C-H, LO-H, and FO-H (*p* < 0.05). The level of linoleic acid was the highest in group FO-H-C and differed from group C-H (*p* < 0.05), while the content of arachidonic acid was the highest in group C-H and differed significantly from group C, as well as all supplemented groups excluding group LO-H (*p* < 0.05) (Table 2). Significant differentiation between the groups was also noted in case of other UFAs. The level of palmitoleic acid (C16:1) was the highest in group C and differed significantly from group C-H, and all supplemented groups (*p* < 0.05). Significant differences were also noted in the case of margarinoleic acid (C17:1), as well as eicosadienoic acid (C20:2) (Table 2).

The summary of UFA levels in individual groups demonstrate the highest level of total UFA in group C, which differed significantly from groups C-H, LO-H, and FO-H (*p* < 0.05), a similar relationship was noted for monounsaturated fatty acid (MUFA) content. No statistically significant differences were observed for the content of polyunsaturated fatty acids (PUFAs), as well as ratios of PUFA/MUFA and PUFA/UFA. The ratio of UFA/SFA was the highest in group C and differed significantly from groups C-H, C-H-C, LO-H, FO-H, as well as FO-H-C (*p* < 0.05) (Table 3).

Considering the content of acids from omega-3 group, the content of alpha-linoleic acid (C18:3n3, ALA) was the highest in group EE-H-C and differed significantly from all other groups (Table 2). An increase in the content of this acid was found in all supplemented groups; compared to the control, it was more than 2-fold higher in group EE-H-C and over 50% higher in group EE-H (Table 2, Figure 1). The content of eicosapentaenoic acid (C20:5n3, EPA) was the highest in group EE-H and it differed significantly from other groups except LO-H-C and EE-H-C (*p* < 0.05). Comparing to the control group C, the level of EPA increased by over 80% in groups EE-H and EE-H-C (Table 2, Figure 1). In the case of docosahexaenoic acid (C22:6n3, DHA), its level was the highest in group FO-H-C and it differed significantly from all other groups (*p* < 0.05). The percentage content compared to the control increased by more than 3-fold in group FO-H-C and over 2-fold in group FO-H (Table 2, Figure 1).

The sum of omega-3 acids was the highest in group EE-H-C and it differed from all control groups and groups LO-H, EE-H, and LO-H-C, while the level of omega-6 acids was the highest in group LO-H-C and differed from groups FO-H, EE-H-C, and FO-H-C (*p* < 0.05). In turn, the ratio of omega-6 to omega-3 was the highest in group LO-H and it was significantly different from other supplemented groups (*p* < 0.05). On the other hand, omega-3 index, i.e., the sum of EPA and DHA acids, was the highest in group FO-H-C and differed significantly from all other groups (*p* < 0.05) (Table 3).

Differences were also noted in the case of atherogenic (AI) and thrombogenic (TI) indices. AI was the highest in group C-H-C and differed significantly from groups C, LO-H-C, and FH-H-C, while TI was the highest in group C-H-C and differed from supplemented groups excluding LO-H (*p* < 0.05) (Table 3).

### 2.3. Fatty Acids Composition of Blood Serum

**Figure 2:**
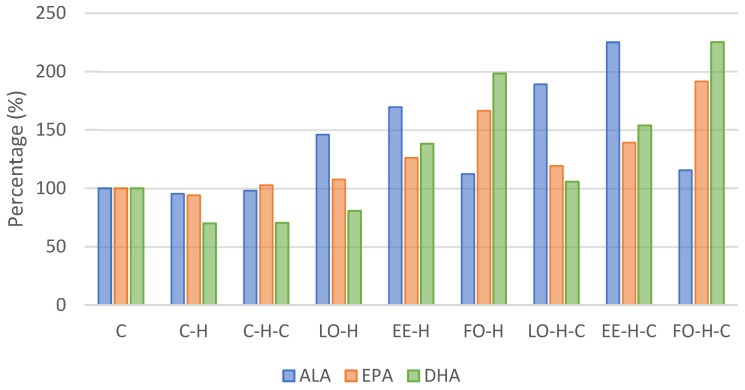
Omega-3 acid content in experimental groups blood serum relative to the control group (C). Explanations of groups–see Table 1; ALA–alpha-linolenic acid; EPA—eicosapentaenoic acid; DHA—docosahexaenoic acid.

Table 4 and Table 5 and Figure 2 present the profile of fatty acids in blood serum of particular groups of animals.

In the case of saturated fatty acids (SFAs), the highest levels were noted for palmitic acid (C16:0) and stearic acid (C18:0) (Table 4). The content of palmitic acid was the highest in group LO-H and differed significantly from all control groups (*p* < 0.05), while the stearic acid level was the highest in group LO-H, and differed from control groups as well as group LO-H-C (*p* < 0.05). Significant differences were also observed in the case of other SFAs. The content of lauric acid (C12:0) was the highest in group FO-H-C and differed from control groups, the myristic acid (C14:0) level was the highest in group LO-H and differed significantly from the control groups and EE-H-C, the tricosanoic acid (C23:0) content was the highest in group EE-H and differed from groups LO-H, FO-H, LO-H-C, and FO-H-C, while the content of lignoceric acid (C24:0) was the highest in group FO-H-C and it was significantly different from control groups as well as groups LO-H and, LO-H-C (*p* < 0.05) (Table 4).

Differentiation in SFA content in individual groups is also reflected in their total content presented in Table 6. It was the highest in group LO-H and differed significantly from control groups and groups LO-H-C and EE-H-C (*p* < 0.05) (Table 5).

Considering unsaturated fatty acids (UFAs), the highest levels were found for oleic acid (C18:1n9c), linoleic acid (C18:2n6c), and arachidonic acid (C20:4n6) (Table 6). The content of oleic acid was the highest in group FO-H and differed significantly from groups C, C-H-C, and EE-H as well as EE-H-C and FO-H-C (*p* < 0.05). The linoleic acid level was the highest in group LO-H and only differed significantly from group LO-H-C (*p* < 0.05). In turn, the arachidonic acid level was the highest in group C and it was significantly different from all other groups except C-H-C (*p* < 0.05). Significant differentiation was also observed in the case of other unsaturated fatty acids. The level of palmitoleic acid (C16:1) was the highest in group C-H-C and differed from all other groups excluding group C. In the case of margarinoleic acid (C17:1) and gamma-linolenic acid (C18:3n6), the highest content was observed in FO-H-C group and it differed from other groups (*p* < 0.05), which was also noted for eicosenic acid (C20:1n9) and eicosadienoic acid (C20:2) (Table 4).

The summary of unsaturated fatty acid content demonstrated the highest level in group LO-H-C and it differed significantly from groups LO-H, EE-H, FO-H, and FO-H-C (*p* < 0.05). The content of monounsaturated fatty acids (MUFAs) was the highest in group FO-H and it differed significantly from other groups except groups C-H, LO-H, and LO-H-C, while the level of polyunsaturated fatty acids (PUFAs) was the highest in group C and it was significantly different from groups C-H, LO-H, FO-H, and FO-H-C (*p* < 0.05). These relationships are reflected in the ratios of particular groups of fatty acids, i.e., PUFA/MUFA as well as PUFA/UFA which were the highest in group C, and differed from C-H, LO-H, and FO-H. In turn, the ratio of UFA/SFA was the highest in group C-H-C and differed significantly from groups LO-H, EE-H, FO-H, and FO-H-C (*p* < 0.05) (Table 5).

Taking into account the levels of acids from the omega-3 group, their level was the highest for ALA, followed by DHA and EPA. Alpha-linolenic acid content was the highest in group EE-H-C and differed significantly from all other groups (*p* < 0.05). Comparing to the control group, the level of this acid increased over 2-fold in group EE-H-C and nearly 90% in group LO-H-C (Figure 2). The content of eicosapentaenoic acid was the highest in group FO-H-C and it was significantly different from other groups except that of group FO-H, and the same was noted for the docosahexaenoic acid content (Table 5). An approximate 80% increase compared to the control was found for EPA in groups EE-H and EE-H-C, and this over 2-fold and 3-fold increase was observed for DHA in groups FO-H and FO-H-C, respectively (Table 4, Figure 2).

The sum of acids from the omega-3 family was the highest in group EE-H-C and it differed significantly from all other groups, while the omega-6 acid level was the highest in group LO-H-C and it differed from group LO-H (*p* < 0.05). This was reflected in the ratio of omega-6 to omega-3 acids, which was the highest in group C-H-C and differed from all supplemented groups (*p* < 0.05) (Table 5).

The atherogenic index value was the highest in group LO-H, and it was significantly different from groups C-H, C-H-C, LO-H-C, and EE-H-C, while the thrombogenic index, also the highest in group LO-H, differed from other supplemented groups (*p* < 0.05) (Table 5).

## 3. Discussion

There are different opinions concerning alpha-linolenic acid’s properties in preventing cardiovascular diseases. Some authors have claimed that this effect is due to an enhanced conversion of ALA to EPA and DHA when the diet is rich in ALA, while other authors have concluded that ALA itself exhibits some health-promoting properties, and therefore it is important to include all n-3 polyunsaturated fatty acids (PUFAs) in the diet [23,24].

In our study, we principally aimed to demonstrate an effect of ethyl esters in linseed oil supplementation on the changes in blood erythrocytes and serum fatty acids profile, emphasizing acids from the omega-3 family. To our knowledge, no studies have been conducted on the effect of such supplements on the fatty acid profile of blood components so far. Linseed oil and fish oil were used for comparative purposes as one of the most commonly used omega-3 supplements.

No significant effect of supplementation on saturated fatty acids (SFAs) between supplemented groups and respective controls were observed in the case of erythrocytes. The groups fed a high-fat diet for the whole experiment (C-H vs. LO-H, EE-H, and FO-H), and groups that changed the diet from a high-fat one to control before the supplementation (C-H-C vs. LO-H-C, EE-H-C, and FO-H-C) did not demonstrate significant differences. However, a decreasing tendency was observed in the supplemented groups compared to the control. A similar observation was made for blood serum; however, some differences in this case were significant—higher total SFAs were noted in the supplemented groups.

Analyzing the total unsaturated fatty acid (UFA) content, a tendency towards an increase was observed in the supplemented groups compared to the respective controls in the case of erythrocytes, and this tendency was more pronounced for groups fed first the high-fat and then the control diets. No clear picture was found for blood serum UFAs.

However, despite the lack of significant differences in total SFAs and UFAs, analysis of particular omega-3 acids confirms the beneficial effect of supplementation on the content of these acids both in erythrocytes and serum. As expected, ALA content in erythrocytes increased in groups supplemented with both linseed oil and ethyl esters. Interestingly, the content of EPA in erythrocytes increased more in groups supplemented with ethyl esters of linseed oil compared to fish oil. This may suggest the process of ALA conversion taking place in these groups. DHA content in erythrocytes also increased with relation to the respective control groups. An important issue that should be kept in mind considering the conversion of ALA to EPA and DHA is the fact of competition between LA (linoleic acid) and ALA. Both acids use the same pathways in the process of long-chain PUFA synthesis and therefore they compete for the same enzymes, i.e., elongases and desaturases [10]. Special importance is attributed to delta-5 desaturase and delta-6 desaturase, which are considered to be the key enzymes in the endogenous synthesis of LC-PUFAs from essential fatty acids [25].

In the study conducted by Young et al. [26], the 12-week supplementation of adults with flax oil and fish oil resulted in about a 10% increase in SFA blood serum content in the fish oil group with no changes in the flaxseed oil group; the UFA level also did not change in the flax oil group and even decreased in the fish oil group. Omega-3 acids in total increased by about 133% in the flax oil group and as much as 187% in the fish oil group; regarding particular omega-3 acids, this change was about 133% for ALA, 155% for EPA, and a 3% decrease for DHA in the case of the flax oil group, and a 33% decrease, 866%, and 112% increase for fish oil, respectively.

The study by Liu et al. [27] applied plant-derived (perilla oil) and marine (fish oil) FA supplements in patients with type 2 diabetes. The authors noted a significant increase in erythrocyte ALA, n-6/n-3, and ALA/EPA levels, and a decrease in EPA, DHA, total n-3 PUFAs, and omega-3 index in the perilla group compared to the fish oil group, which is consistent with our results in terms of the differences between plant and animal omega-3 sources.

In the study by Barceló-Coblijn et al. [28], adult participants were supplemented for 12 weeks with different doses of flaxseed oil and fish oil. The authors observed that the ALA content increased significantly in groups supplemented with flaxseed oil, regardless of the dose. By the end of the study, the authors noted a slight tendency of a decrease in ALA which they suggested could have been a result of the ALA conversion to EPA and further to DHA. ALA content increase in fish oil-supplemented groups was not significant. The increase in the EPA and DHA content in flaxseed oil-supplemented group was not significant, while in case of fish oil it increased by about 113 and 40%, respectively. The study by Leung et al. [23] demonstrated that supplementation of rats’ diet with flaxseed and flaxseed oil increased ALA and EPA levels in blood plasma, but did not change DHA level, and reduced omega-6 PUFA content, i.e., AA and AdA. This suggested that the diet enriched in ALA caused an increased conversion of ALA to EPA and DHA, and a reduced conversion of AA to AdA; however, it also confirmed poor ALA conversion to DHA [23].

The dietary intake of fatty acids is in part reflected by the profile of fatty acids of various organs and tissues. In the case of blood serum and erythrocyte membranes, it is suggested that it reflects the intake of several weeks. However, the fatty acid profile in blood compounds, as well as other biological tissues, to a significant degree depends on their endogenous metabolism which probably may also differ depending on the source of fatty acids in the diet [2,29,30]. Other reports in the literature suggest, in turn, that the fatty acid content in erythrocytes has some advantages over their profile in serum including longer half-life, presence of lipid bilayer, and lack of lipoproteins increasing the variation in FA profile [31,32]. In turn, according to Hu et al. [33], the ratios of FA conversion from plasma to erythrocytes are quite stable.

The main limitation in our study is the small number of animals in any particular study group. Other limitations may include the duration of the supplementation, the specific method of fatty acid analysis, or potential confounding factors like baseline dietary habits or genetic differences between subjects. Additionally, it would be reasonable to examine other parameters involved in the mechanism of alpha-linolenic acid conversion and factors relating to this phenomenon, which require further studies.

## 4. Materials and Methods

### 4.1. Animals and Scheme of the Experiment

**Figure 3:**
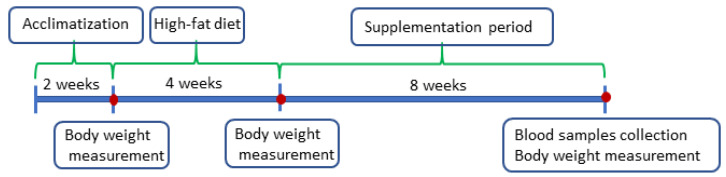
Scheme of the experiment.

This study was carried out on male Wistar rats obtained from the monozygotic Charles Rivers Laboratories (Germany). The rats were maintained individually in the vivarium of the Faculty of Veterinary Medicine, Wrocław University of Environmental and Life Sciences, Poland, at a temperature of about 21 °C with a 12 h light/dark cycle. They were randomly divided into 9 groups (simple randomization–random number generation), with eight animals in each group. The rats were fed the control diet (C) with w/10% energy from fat (diet no. C 1090-10; https://altromin.com/products/specialdiets/highfatdiets/C1090-10; accessed on 20 December 2024) and high-fat diet (H) with w/70% energy from fat (42% fat) (diet no. C1090-70; https://altromin.com/products/specialdiets/highfatdiets/C1090-70; accessed on 20 December 2024) from Altromin International (Germany), and had ad libitum access to water. The scheme of supplementation is presented in Table 6, and the scheme of the experiment in Figure 3. 

Prior to the experiment, the rats were subjected to 2 weeks acclimatization period, followed by a 4 week period when the animals (excluding control group) were fed a high-fat diet. The experiment lasted 8 weeks; during this time, the animals from the experimental groups were supplemented with linseed oil (LO) which was the raw material for the production of ethyl esters, linseed oil ethyl esters (EE), and fish oil (FO). All supplements were administered orally using a syringe in amount of 0.04 g/kg body weight per day. Body weight of the animals was controlled during the experiment.

### 4.2. Supplements

Synthesis of ethyl esters of linseed oil was conducted according to the technology developed at the University of Wrocław (Poland) [34]. The production technology and characteristics of ethyl ester used in this experiment are presented in the study by Sokoła-Wysoczańska et al. [10]. In brief, the technology involves transesterification of linseed oil (a mixture of triglycerides of omega-3, -6, -9 fatty acids) with ethanol in the presence of a catalyst. The first stage of the process involves transesterification in an anaerobic atmosphere, then unreacted bioethanol is removed from the post-reaction mixture and the glycerin phase is separated from the raw ester phase in gravity separators. Then, the raw esters were cleaned by centrifugation, followed by cleaning using residual gas alcohol depot with nitrogen, and the residual glycerin phase was subjected to sedimentation. The glycerin phase is separated in the last step of the process.

For comparative purposes, we also applied raw linseed oil, which was a substrate for ethyl esters synthesis, and commercially available fish oil (cod liver).

The fatty acid profiles of supplements applied in this study are presented in Table 7.

### 4.3. Blood Sampling and Analyses

On the last day of the experiment, all animals were euthanized and blood samples were collected to determine the fatty acid profile in erythrocytes and blood serum. The blood was centrifuged at 1000 rpm, separated into serum and morphotic elements, and then stored at −80 °C. Fat present in serum and erythrocytes was extracted by Folch method.

Blood serum and erythrocyte samples were prepared according to the method described by Kroger et al. [35], and fatty acid methyl esters (FAMEs) were prepared according to the method elaborated by Prescha et al. [36]. FAME analysis was performed using a 6890 N gas chromatograph (Agilent Technologies, Santa Clara, CA, USA) equipped with a flame ionization detector (FID) and an Rtx 2330 100 m × 0.25 mm × 0.5 mm capillary column (Restek, Bellefonte, PA, USA). Hydrogen at a flow rate of 1.5 mL/min was used as the carrier gas and the separation was carried out at a temperature range from 165 °C (for 10 min) to 220 °C, with an increase rate of 2 °C/min. Individual fatty acids were identified by comparison of sample peak retention times with FAME standard mixture (Merck Life Science Ltd., Poznań, Poland). Pentadecanoic acid was used as an internal standard for quantitative analysis and Chemstation vB.04.02 (Agilent Technologies, Santa Clara, CA, USA) was used to calculate the results.

Data on fatty acids are presented as a percentage of individual acids in the total acid pool. Total share of saturated acids (SFAs), unsaturated fatty acids (UFAs), monounsaturated fatty acids (MUFAs), and polyunsaturated fatty acids (PUFAs), as well as their ratios, were calculated. In addition, the content of total n-3, n-6, and n-9 fatty acids was determined. Omega-3 index was calculated as the sum of eicosapentaenoic and docosahexaenoic acids in erythrocytes [31].

The lipid quality indices, i.e., atherogenic index (AI) and thrombogenic index (TI), were calculated based on the fatty acid profiles of examined samples according to the following formulas [37,38]:

AI = (C12:0 + 4 × C14:0 + C16:0)/(n-6 PUFA + n-3 PUFA + MUFA)

TI = (C14:0 + C16:0 + C18:0)/(0.5 × n-6 PUFA + 3 × n-3 PUFA + n-3 PUFA/n-6 PUFA).

This study was carried out with the agreement of the 2nd Local Animal Ethics Committee, Wrocław University of Environmental and Life Sciences, Poland (approval no. 79/2010).

### 4.4. Statistical Analysis

The results were analyzed statistically using Statistica 13.0 (StatSoft, Krakow, Poland) and presented as mean values and standard deviation (SD). The normality of distribution was examined using the Shapiro–Wilk test. One-factor Anova was conducted, the significance of differences between the groups was determined using Tukey’s test at the significance level of *p* < 0.05.

## 5. Conclusions

Despite some variations in the levels of individual fatty acids in erythrocytes and the serum of individual groups supplemented with various sources of fatty acids, our results indicate that a more profitable FA profile, considering, among others, the content of omega-3 acids, n6/n3 ratio, or omega-3 index, was obtained for groups fed first a high-fat diet and then a control diet during the supplementation period. This may suggest the importance of supplementation in combination with beneficial dietary patterns changes. More beneficial results in this regard were also obtained in the groups supplemented with ethyl esters of linseed oil, compared to linseed oil, which indicates the improved bioavailability of this supplement, and justify further research in this range.

## Figures and Tables

**Table 1:** Body weight of the experimental animals during the experiment.

		Groups								
		C	C-H	C-H-C	LO-H	EE-H	FO-H	LO-H-C	EE-H-C	FO-H-C
After acclimatization	mean	378.5	384.8	374.0	388.0	371.8	378.3	381.7	392.2	392.8
	SD	9.6	17.8	12.3	22.4	14.1	17.8	22.5	11.2	14.8
Beginning of supplementation	mean	460.3	473.2	439.5	445.0	435.5	456.5	459.0	433.5	450.7
	SD	15.0	38.5	17.3	45.1	27.9	27.1	34.6	13.2	14.1
End of the experiment	mean	575.7 ^b^	651.3 ^a^	518.3 ^d^	553.0 ^b,d^	587.8 ^b,c^	630.0 ^a,c^	580.5 ^b^	571.5 ^b^	587.3 ^b,c^
	SD	16.2	25.4	35.3	25.6	20.4	17.8	26.0	18.3	20.6

^a–d^ different superscripts indicate statistical differences between the groups at *p* < 0.05.

**Table 2:** Fatty acid profile in erythrocytes (% of total fatty acids).

Fatty Acid		Group								
		C	C-H	C-H-C	LO-H	EE-H	FO-H	LO-H-C	EE-H-C	FO-H-C
Saturated fatty acids										
Lauric acid C12:0	X	0.284 ^b,c^	0.476 ^a^	0.357 ^a,c^	0.353 ^a,c^	0.400 ^a,c^	0.289 ^b,c^	0.305 ^b,c^	0.341 ^a,c^	0.273 ^b,c^
	SD	0.060	0.134	0.107	0.086	0.098	0.035	0.071	0.076	0.088
Myristic acid C14:0	X	1.003	1.271	1.216	1.308	1.376	1.264	0.995	1.227	0.996
	SD	0.477	0.093	0.302	0.082	0.100	0.167	0.208	0.453	0.227
Palmitic acid C16:0	X	23.260 ^b,c^	26.024 ^a,c^	27.485 ^a^	25.422 ^a,c^	23.690 ^b,c^	25.763 ^a,c^	23.814 ^b,c^	24.842 ^a,c^	23.128 ^b,c^
	SD	1.947	1.959	1.494	1.864	1.260	1.582	1.065	2.464	2.444
Margaric acid C17:0	X	0.390 ^b,c,d,e^	0.599 ^a^	0.491 ^a,e^	0.596 ^a^	0.525 ^a,c^	0.599 ^a^	0.440 ^b,c,d,e^	0.492 ^a,d^	0.384 ^b,d,e^
	SD	0.078	0.079	0.083	0.043	0.037	0.089	0.066	0.149	0.082
Stearic acid C18:0	X	9.679 ^b,c,d^	10.817 ^a,c^	10.229 ^a,d^	11.601 ^a^	10.676 ^a,d^	10.717 ^a,d^	9.929 ^b,c,d^	9.608 ^b,c,d^	8.647 ^b^
	SD	1.003	1.001	0.633	0.970	0.844	1.040	0.887	0.763	0.907
Arachidic acid C20:0	X	0.418 ^a,c^	0.275 ^b,c,d^	0.244 ^b,d^	0.304 ^a,d^	0.347 ^a,d^	0.324 ^a,d^	0.327 ^a,d^	0.417 ^a,c^	0.464 ^a^
	SD	0.093	0.065	0.054	0.054	0.067	0.081	0.096	0.069	0.223
Behenic acid C22:0	X	0.275 ^b^	0.321 ^b^	0.291 ^b^	0.336 ^b^	0.238 ^b^	0.279 ^b^	0.866 ^a^	0.227 ^b^	0.656 ^a^
	SD	0.123	0.144	0.087	0.160	0.041	0.079	0.333	0.067	0.247
Tricosanoic acid C23:0	X	0.763	0.474 ^a^	0.327 ^a^	0.478 ^a^	0.649	0.414 ^a^	0.646	0.830	0.993 ^b^
	SD	0.439	0.059	0.037	0.147	0.249	0.346	0.286	0.497	0.408
Lignoceric acid C24:0	X	0.188 ^e^	0.329 ^e^	0.256 ^e^	0.943 ^b,d^	1.168 ^a,d^	1.755 ^a^	1.109 ^b,c,d^	1.559 ^a,c^	1.068 ^b,c,d^
	SD	0.110	0.083	0.027	0.212	0.402	0.352	0.332	0.849	0.344
Unsaturated fatty acids										
Palmitoleic acid C16:1	X	1.117 ^a^	0.821 ^d,e,g^	1.065 ^a,c^	0.561 ^f,h^	0.762 ^e,g,h^	0.651 ^e,g,h^	0.721 ^e,g,h^	0.866 ^b,c,e^	0.842 ^c,g^
	SD	0.098	0.104	0.094	0.082	0.141	0.082	0.088	0.130	0.313
Margarinoleic acid C17:1	X	0.998	1.123 ^a^	1.061 ^a^	1.118 ^a^	0.826	0.904	0.937	0.889	0.664 ^b^
	SD	0.205	0.149	0.083	0.138	0.121	0.358	0.346	0.223	0.296
Oleic acid C18:1n9	X	25.747 ^a^	20.421 ^b^	20.886	20.111 ^b^	23.598	20.545 ^b^	22.327	22.509	23.230
	SD	4.421	2.056	1.711	2.699	2.724	1.850	2.793	4.788	3.520
Linoleic acid C18:2n6	X	19.529	16.626 ^a^	17.105	18.408	19.968	18.016	20.969 ^b^	19.074	20.994 ^b^
	SD	3.245	2.431	1.989	1.488	2.991	2.841	1.963	2.961	2.211
Alpha-linolenic acid C18:3n3	X	1.852 ^c,d,e,f,g^	1.740 ^c,d,e,f,g^	1.751 ^c,d,e,f,g^	2.284 ^b,e^	2.909 ^b^	2.502 ^b,d^	2.171 ^b,g^	4.108 ^a^	2.202 ^b,f^
	SD	0.496	0.487	0.478	0.478	0.855	1.023	0.471	0.810	0.204
Eicosenic acid C20:1n9	X	0.606	0.569	0.576	0.635	0.779	0.925	0.613	0.852	0.957
	SD	0.130	0.244	0.140	0.247	0.260	0.356	0.225	0.349	0.627
c Eicosadienoic acid C20:2	X	0.206 ^a,c^	0.218 ^a,c^	0.265	0.246	0.252	0.300 ^b^	0.270	0.274	0.216 ^c^
	SD	0.016	0.038	0.032	0.023	0.035	0.075	0.047	0.076	0.066
Arachidonic acid C20:4n6	X	10.649 ^b,c,d^	14.033 ^a^	13.523 ^a,c^	11.818 ^a,d^	8.751 ^b,d,f^	9.303 ^b,d,e^	10.485 ^b,c,d^	8.351 ^b,f^	6.747 ^e,f^
	SD	3.152	2.104	1.392	0.913	1.453	1.475	2.134	2.080	2.000
Eicosapentaenoic acid C20:5n3	X	0.305 ^c^	0.228 ^c^	0.237 ^c^	0.314 ^c^	0.552 ^a^	0.305 ^c^	0.351 ^a,c^	0.550 ^a^	0.318 ^b,c^
	SD	0.114	0.032	0.035	0.077	0.208	0.058	0.142	0.232	0.050
Docosahexaenoic acid C22:6n3	X	1.292 ^d,e,f^	1.270 ^d,e,f^	1.211 ^d^	1.828 ^c,e^	1.826 ^c,f^	2.866 ^b^	2.099 ^c^	2.063 ^c^	3.959 ^a^
	SD	0.350	0.106	0.250	0.327	0.287	0.625	0.280	0.193	0.523
Nervonic acid C24:1	X	0.152 ^c,e^	0.554 ^a,c^	0.625 ^a^	0.661 ^a^	0.117 ^c,f,g^	0.508 ^a,e^	0.198 ^b,c,d,e,f^	0.185 ^c,d,e,g^	0.522 ^a,d^
	SD	0.054	0.158	0.178	0.288	0.067	0.260	0.134	0.074	0.455
other	X	1.287	1.810	0.799 ^a^	0.676 ^a^	0.591 ^a^	1.772	0.428 ^a^	0.736 ^a^	2.737 ^b^
	SD	0.929	0.704	0.538	0.256	1.036	1.183	0.265	0.496	2.797

^a–h^ Different superscripts indicate statistical differences between the groups at *p* < 0.05.

**Table 3:** Summary of fatty acid content in erythrocytes (% of total fatty acids).

Parameter		Group								
		C	C-H	C-H-C	LO-H	EE-H	FO-H	LO-H-C	EE-H-C	FO-H-C
Total SFA	X	36.259 ^a,d^	40.586	40.896	41.341 ^b^	39.069	41.404 ^b^	38.431	39.544	36.611 ^c,d^
	SD	3.041	2.872	1.759	2.801	2.074	2.595	1.604	4.382	3.722
Total UFA	X	62.454 ^a^	57.604 ^b^	58.304	57.983 ^b^	60.34	56.824 ^b^	61.141	59.72	60.652
	SD	2.795	2.65	1.89	2.843	2.051	3.047	1.718	4.039	2.404
Total MUFA	X	28.621 ^a^	23.489 ^b^	24.212	23.085 ^b^	26.083	23.532 ^b^	24.795	25.301	26.216
	SD	4.155	2.154	1.537	2.954	2.792	1.909	2.946	4.371	3.443
Total PUFA	X	33.832	34.115	34.092	34.898	34.258	33.292	36.346	34.42	34.436
	SD	2.43	1.121	1.456	1.455	3.399	2.916	2.268	3.243	3.064
PUFA/MUFA	X	1.209	1.462	1.414	1.534	1.334	1.425	1.491	1.407	1.344
	SD	0.22	0.131	0.118	0.209	0.235	0.192	0.256	0.328	0.27
PUFA/UFA	X	0.543	0.593	0.585	0.603	0.567	0.585	0.595	0.578	0.568
	SD	0.05	0.022	0.021	0.034	0.049	0.032	0.041	0.057	0.051
UFA/SFA	X	1.737 ^a,c^	1.430 ^b,d^	1.430 ^b,d^	1.413 ^b,d^	1.55	1.381 ^b^	1.595	1.536	1.676 ^c,d^
	SD	0.206	0.173	0.11	0.172	0.133	0.165	0.112	0.27	0.218
Total n3	X	3.449 ^e,g^	3.238 ^e^	3.198 ^e^	4.426 ^b,d,f,g^	5.287 ^b,c,d^	5.673 ^a,c^	4.622 ^b,c,f^	6.721 ^a^	6.479 ^a^
	SD	0.641	0.553	0.62	0.434	0.698	1.193	0.338	0.658	0.558
Total n6	X	30.177	30.659	30.628	30.226	28.718	27.319 ^a^	31.454 ^b^	27.425 ^a^	27.741 ^a^
	SD	2.026	0.854	1.35	1.165	3.494	1.986	2.058	2.871	2.877
Total n9	X	26.354 ^a^	20.991 ^b^	21.462	20.745 ^b^	24.377	21.47	22.94	23.361	24.188
	SD	4.331	2.288	1.621	2.832	2.79	1.796	2.847	4.557	3.348
n6/n3	X	9.031 ^a^	9.709 ^a^	9.861 ^a^	6.876 ^b^	5.529 ^b,d^	4.954 ^c,d^	6.825 ^b^	4.098 ^c,d^	4.304 ^c,d^
	SD	1.808	1.68	1.709	0.585	1.106	0.815	0.5	0.411	0.56
AA/EPA	X	38.682 ^b,c,f^	63.627 ^a^	58.142 ^a,c^	40.200 ^b,c,d^	19.082 ^e,f,h,i^	31.565 ^b,d,h^	33.581 ^b,d,g^	17.585 ^e,g,h,i^	22.287 ^b,d,i^
	SD	16.264	18.274	9.492	12.776	11.908	7.892	13.454	7.855	8.757
Omega-3 index	X	1.597 ^e,f^	1.498 ^e^	1.448 ^e^	2.142 ^c,d,f^	2.378 ^c,d^	3.171 ^b^	2.451 ^c,d^	2.613 ^b,d^	4.277 ^a^
	SD	0.384	0.107	0.265	0.292	0.272	0.629	0.349	0.37	0.53
AI	X	0.445 ^b,d,e,f^	0.553 ^a,c^	0.565 ^a^	0.539 ^a,d^	0.493 ^a,f^	0.553 ^a,c^	0.462 ^b,c,d,e,f^	0.512 ^a,e^	0.455 ^b,d,e,f^
	SD	0.056	0.06	0.056	0.056	0.034	0.057	0.031	0.1	0.06
TI	X	1.339 ^a,d^	1.529 ^a^	1.568 ^a^	1.347 ^a,c^	1.184 ^b,c,d,f^	1.250 ^b,c,d^	1.171 ^b,c,d,g^	1.055 ^b,h^	0.978 ^e,f,g,h^
	SD	0.153	0.214	0.173	0.128	0.137	0.232	0.082	0.155	0.081

^a–i^ Different superscripts indicate statistical differences between the groups at *p* < 0.05; SFA—saturated fatty acids; UFA—unsaturated fatty acids; MUFA—monounsaturated fatty acids; PUFA—polyunsaturated fatty acids; AA–arachidonic acid; EPA—eicosapentaenoic acid; AI—atherogenic index; TI—thrombogenic index.

**Table 4:** Fatty acid profile in blood serum (% of total fatty acids).

		Group								
Fatty Acid		C	C-H	C-H-C	LO-H	EE-H	FO-H	LO-H-C	EE-H-C	FO-H-C
Saturated fatty acids										
Lauric acid C12:0	X	0.462 ^a^	0.511 ^a,c^	0.527 ^a,c^	0.638 ^b,c^	0.627 ^b,c^	0.624 ^b,c^	0.619 ^b,c^	0.590	0.685 ^b^
	SD	0.056	0.067	0.084	0.057	0.088	0.064	0.084	0.069	0.146
Myristic acid C14:0	X	1.918 ^d,e^	1.959 ^c,e^	1.859 ^d,e^	2.592 ^a^	2.472 ^a^	2.199 ^a,e^	2.219 ^a,e^	2.059 ^b,c,e^	2.348 ^a,c^
	SD	0.160	0.374	0.139	0.239	0.365	0.288	0.233	0.153	0.242
Palmitic acid C16:0	X	19.743	19.218 ^a^	19.427 ^a^	21.316 ^b^	20.461	20.072	19.614	19.769	20.429
	SD	0.717	1.100	0.695	0.686	1.213	1.413	1.225	1.274	1.636
Margaric acid C17:0	X	0.431	0.463	0.386	0.533	0.550 ^a^	0.419	0.357 ^b^	0.427	0.378
	SD	0.078	0.064	0.049	0.072	0.098	0.120	0.212	0.059	0.167
Stearic acid C18:0	X	6.274 ^c,d,f^	6.482 ^c,d,f^	5.878 ^b,e,f^	7.833 ^a^	7.611 ^a^	7.654 ^a^	6.518 ^b,c,d,e^	6.913 ^a,d^	7.258 ^a,c^
	SD	0.214	0.306	0.402	0.321	0.585	0.441	0.601	0.829	1.250
Arachidic acid C20:0	X	0.474 ^a^	0.509	0.446 ^a^	0.484	0.487	0.528	0.605	0.458 ^a^	0.749 ^b^
	SD	0.117	0.062	0.079	0.065	0.085	0.061	0.241	0.140	0.366
Behenic acid C22:0	X	0.335	0.367	0.302 ^a^	0.348	0.330	0.307 ^a^	0.357	0.273 ^a^	0.475 ^b^
	SD	0.086	0.038	0.031	0.048	0.052	0.067	0.056	0.055	0.227
Tricosanoic acid C23:0	X	1.071 ^a,f^	1.348 ^a,c^	1.229 ^a,e^	1.012 ^c,e,f^	1.639 ^a^	0.672 ^d,e,f^	0.902 ^c,e,f^	1.129 ^a,f^	1.029 ^b,c,e,f^
	SD	0.305	0.280	0.346	0.207	0.719	0.091	0.211	0.498	0.134
Lignoceric acid C24:0	X	0.284 ^g,h^	0.229 ^h^	0.345 ^e,f,h^	0.413 ^b,d,h^	0.874 ^a,c^	0.667 ^a,d^	0.607 ^b,c,d,f^	0.830 ^a,c^	0.935 ^a^
	SD	0.029	0.073	0.049	0.083	0.304	0.213	0.156	0.259	0.239
Unsaturated fatty acids										
Palmitoleic acid C16:1	X	1.981 ^a,c^	1.566 ^c,f,g^	2.532 ^a^	1.180 ^b,d,f,h^	0.978 ^e,g,h^	1.319 ^c,f,g^	1.499 ^c,f,g^	1.700 ^b,c,d^	1.698 ^c,f^
	SD	0.482	0.183	0.880	0.204	0.161	0.235	0.386	0.459	0.147
Margarinoleic acid C17:1	X	0.137 ^a^	0.172	0.148 ^a^	0.153 ^a^	0.124 ^a^	0.135 ^a^	0.128 ^a^	0.129 ^a^	0.238 ^b^
	SD	0.028	0.026	0.030	0.013	0.038	0.037	0.022	0.019	0.105
Oleic acid C18:1n9	X	26.718 ^b,d,e,f^	30.725 ^a^	28.496 ^b,c,d^	31.979 ^a^	27.155 ^b,d,e,f^	32.020 ^a^	29.754 ^a,c^	28.315 ^c,e^	27.698 ^c,f^
	SD	1.070	1.416	0.452	1.342	1.602	1.226	1.989	1.934	1.431
Linoleic acid C18:2n6	X	23.995	23.423	25.312	22.569 ^a^	25.040	23.396	26.246 ^b^	24.893	24.173
	SD	1.688	2.156	2.659	1.531	2.578	2.231	2.407	1.286	1.248
Alpha-linolenic acid C18:3n3	X	2.341 ^e,f^	2.239 ^e,f^	2.301 ^e,f^	3.423 ^c,d^	3.976 ^b,d^	2.633 ^e,f^	4.434 ^b^	5.273 ^a^	2.712 ^c,f^
	SD	0.576	0.487	0.451	0.388	0.614	0.290	0.504	0.627	0.191
Gamma-linolenic acid C18:3n6	X	0.242	0.209 ^a^	0.237 ^a^	0.200 ^a^	0.223 ^a^	0.195 ^a^	0.238 ^a^	0.193 ^a^	0.316 ^b^
	SD	0.064	0.050	0.040	0.076	0.042	0.029	0.046	0.025	0.042
Eicosenic acid C20:1n9	X	0.993	1.094	0.722 ^a^	1.010	1.127 ^b^	0.985	1.100	0.929	1.206 ^b^
	SD	0.191	0.093	0.148	0.118	0.477	0.183	0.178	0.206	0.329
Eicosadienoic acid C20:2	X	0.287	0.241	0.245	0.194 ^a^	0.233 ^a^	0.237 ^a^	0.291	0.266	0.341 ^b^
	SD	0.044	0.040	0.044	0.033	0.035	0.057	0.105	0.029	0.119
Arachidonic acid C20:4n6	X	8.094 ^a^	4.742 ^b^	5.444 ^a^	2.010 ^d,e,f^	3.413 ^c^	1.937 ^d,f^	2.131 ^d,e,f^	3.001 ^c,e^	2.403 ^c,f^
	SD	0.696	1.047	0.914	0.199	0.515	0.257	0.483	0.715	0.435
Eicosapentaenoic acid C20:5n3	X	0.231 ^e^	0.218 ^e^	0.238 ^e^	0.249 ^e^	0.292 ^c,e^	0.385 ^a,c^	0.276 ^d,e^	0.322 ^b,c,e^	0.443 ^a^
	SD	0.042	0.018	0.038	0.055	0.060	0.067	0.041	0.022	0.156
Docosahexaenoic acid C22:6n3	X	1.117 ^c^	0.782 ^c^	0.788 ^c^	0.902 ^c^	1.543 ^b,d^	2.215 ^a^	1.181 ^c,d^	1.717 ^b^	2.514 ^a^
	SD	0.129	0.227	0.133	0.150	0.375	0.324	0.275	0.208	0.393
other	X	2.870 ^a,c^	3.504 ^a^	3.141 ^a,c^	0.960 ^b^	0.845 ^b^	1.402 ^b^	0.925 ^b^	0.814 ^b^	1.974 ^b,c^
	SD	1.196	1.030	1.088	0.348	0.412	0.690	0.474	0.521	1.077

^a–h^ Different superscripts indicate statistical differences between the groups at *p* < 0.05.

**Table 5:** Summary of fatty acid content in blood serum (% of total fatty acids).

Parameter		Group								
		C	C-H	C-H-C	LO-H	EE-H	FO-H	LO-H-C	EE-H-C	FO-H-C
Total SFA	X	30.992 ^d^	31.086 ^d^	30.398 ^b,d^	35.169 ^a^	35.050 ^a^	33.142 ^a,d^	31.797 ^b,d^	32.448 ^b,c,d^	34.285 ^a,c^
	SD	0.401	1.607	0.792	0.982	2.214	2.043	1.066	1.038	1.597
Total UFA	X	66.137 ^a^	65.410 ^a,e^	66.461 ^a,c^	63.871 ^b,d,e^	64.105 ^b,c,e^	65.456 ^a,e^	67.278 ^a^	66.738 ^a^	63.741 ^b,c^
	SD	1.141	1.682	1.185	1.081	2.320	1.757	1.151	1.272	1.256
Total MUFA	X	29.830 ^b,f,g,h^	33.556 ^a,d^	31.897 ^b,c,d,e,f^	34.323 ^a,c^	29.385 ^f,g,h^	34.459 ^a^	32.481 ^a,e^	31.072 ^b,e,g^	30.839 ^e,h^
	SD	1.530	1.431	0.879	1.356	1.481	1.302	2.161	1.770	1.445
Total PUFA	X	36.307 ^a^	31.854 ^b,d,e,f^	34.564 ^a,e^	29.548 ^f^	34.720 ^a,c,d^	30.997 ^b,f^	34.797 ^a,c,d^	35.665 ^a,c^	32.901 ^b,c,e^
	SD	1.491	2.350	1.737	1.815	2.311	2.023	2.195	0.947	1.360
PUFA/MUFA	X	1.222 ^a^	0.953 ^b,c,d^	1.085 ^a,c^	0.864 ^b^	1.185 ^a^	0.902 ^b^	1.079 ^a,c^	1.152 ^a^	1.070 ^a,d^
	SD	0.104	0.103	0.079	0.084	0.112	0.081	0.134	0.093	0.083
PUFA/UFA	X	0.549 ^a^	0.487 ^b,c^	0.520 ^a,c^	0.462 ^b^	0.541 ^a^	0.473 ^b^	0.517 ^a,c^	0.535 ^a^	0.516 ^a,c^
	SD	0.021	0.027	0.018	0.024	0.023	0.023	0.031	0.020	0.020
UFA/SFA	X	2.134 ^a,c^	2.111 ^a,c^	2.188 ^a^	1.818 ^b^	1.839 ^b^	1.984 ^b,c^	2.119 ^a,c^	2.060 ^a,c,d^	1.864 ^b,d^
	SD	0.047	0.151	0.083	0.081	0.186	0.170	0.103	0.103	0.118
Total n3	X	3.689 ^e^	3.239 ^e^	3.326 ^e^	4.575 ^c,d^	5.810 ^b^	5.233 ^b,d^	5.891 ^b^	7.313 ^a^	5.669 ^b^
	SD	0.554	0.420	0.456	0.463	0.545	0.502	0.383	0.709	0.415
Total n6	X	24.988	24.516	26.033	23.580 ^a^	26.167	24.381	27.346 ^b^	25.822	25.378
	SD	1.580	2.075	2.723	1.619	2.552	2.315	2.403	1.303	1.253
Total n9	X	27.712 ^b,d,e^	31.819 ^a^	29.218 ^c,d^	32.990 ^a^	28.282 ^b,d,e^	33.005 ^a^	30.854 ^a,c^	29.244 ^b,c^	28.903 ^c,e^
	SD	1.152	1.499	0.532	1.275	1.605	1.219	1.908	1.968	1.515
n6/n3	X	6.900 ^a^	7.681 ^a^	7.984 ^a^	5.183 ^b^	4.552 ^b,c^	4.705 ^b,c^	4.655 ^b,c^	3.569 ^c^	4.493 ^b,c^
	SD	1.054	1.157	1.564	0.445	0.738	0.710	0.459	0.480	0.325
AA/EPA	X	36.313 ^a^	21.955 ^b^	23.880 ^b^	8.475 ^c^	12.152 ^c^	5.215 ^c^	7.910 ^c^	9.351 ^c^	6.330 ^c^
	SD	8.237	5.895	7.240	2.175	3.012	1.349	2.329	2.330	3.233
AI	X	0.477 ^a,d^	0.450 ^b,c,d^	0.448 ^c,d^	0.518 ^a^	0.507 ^a,c^	0.461 ^a,d^	0.443 ^d^	0.446 ^b,d^	0.493 ^a,d^
	SD	0.018	0.045	0.025	0.033	0.058	0.046	0.027	0.036	0.033
TI	X	1.188 ^a,c^	1.256 ^a^	1.180 ^a,c^	1.242 ^a^	0.997 ^b,f^	1.068 ^b,c^	0.901 ^d,f,g^	0.821 ^e,g^	1.007 ^b,c^
	SD	0.125	0.110	0.105	0.115	0.076	0.101	0.057	0.061	0.094

^a–h^ Different superscripts indicate statistical differences between the groups at *p* < 0.05; SFAs—saturated fatty acids; UFAs—unsaturated fatty acids; MUFAs—monounsaturated fatty acids; PUFAs—polyunsaturated fatty acids; AA–arachidonic acid; EPA—eicosapentaenoic acid; AI—atherogenic index; TI—thrombogenic index.

**Table 6:** Scheme of supplementation.

	Diet		Supplementation Weeks 5–12
Group	Weeks 1–4	Weeks 5–12	
C	control	control	-
C-H	high-fat	high-fat	-
C-H-C	high-fat	control	-
LO-H	high-fat	high-fat	Linseed oil
EE-H	high-fat	high-fat	Ethyl esters
FO-H	high-fat	high-fat	Fish oil
LO-H-C	high-fat	control	Linseed oil
EE-H-C	high-fat	control	Ethyl esters
FO-H-C	high-fat	control	Fish oil

**Table 7:** Fatty acid profiles of supplements used in this study (% of total fatty acids) [5].

Acid	LO	EE	FO
Palmitic acid-C16:0	4.37	4.44	11.36
Stearic acid-C18:0	3.79	3.43	2.68
Oleic acid-C18:1	16.41	16.73	23.95
Linoleic acid-C18:2	16.24	16.68	1.43
Alpha-linolenic acid-C18:3	56.29	58.71	−
Eicosapentaenoic acid-C20:5	−	−	8.13
Docosahexaenoic acid-C22:6	−	−	9.87

LO—linseed oil; EE—linseed oil ethyl esters; FO—fish oil.

## Data Availability

Data is contained within the article.
